# Roadmap of Adversarial Machine Learning in Internet of Things-Enabled Security Systems

**DOI:** 10.3390/s24165150

**Published:** 2024-08-09

**Authors:** Yasmine Harbi, Khedidja Medani, Chirihane Gherbi, Zibouda Aliouat, Saad Harous

**Affiliations:** 1 LRSD Laboratory, Ferhat Abbas University Setif-1, Setif 19000, Algeria; yasmine.harbi@univ-setif.dz (Y.H.); k.medani@univ-setif2.dz (K.M.); chirihane.gherbi@univ-setif.dz (C.G.); zaliouat@univ-setif.dz (Z.A.); 2 Arabic Literature and Language Department, Mohamed Lamine Debaghine University Setif-2, Setif 19000, Algeria; 3 College of Computing and Informatics, University of Sharjah, Sharjah 27272, United Arab Emirates

**Keywords:** artificial intelligence, IoT, cyberattacks, AML, security defense, systematic literature review

## Abstract

Machine learning (ML) represents one of the main pillars of the current digital era, specifically in modern real-world applications. The Internet of Things (IoT) technology is foundational in developing advanced intelligent systems. The convergence of ML and IoT drives significant advancements across various domains, such as making IoT-based security systems smarter and more efficient. However, ML-based IoT systems are vulnerable to lurking attacks during the training and testing phases. An adversarial attack aims to corrupt the ML model’s functionality by introducing perturbed inputs. Consequently, it can pose significant risks leading to devices’ malfunction, services’ interruption, and personal data misuse. This article examines the severity of adversarial attacks and accentuates the importance of designing secure and robust ML models in the IoT context. A comprehensive classification of adversarial machine learning (AML) is provided. Moreover, a systematic literature review of the latest research trends (from 2020 to 2024) of the intersection of AML and IoT-based security systems is presented. The results revealed the availability of various AML attack techniques, where the Fast Gradient Signed Method (FGSM) is the most employed. Several studies recommend the adversarial training technique to defend against such attacks. Finally, potential open issues and main research directions are highlighted for future consideration and enhancement.

## 1. Introduction

Internet-connected systems are spreading in all aspects of our society that rely on critical infrastructure, such as manufacturing industries, hospitals, educational institutions, financial companies, etc. Several protocols, frameworks, and technologies have emerged to meet the increasing needs of citizens. However, the widespread penetration of these solutions is faced by several security threats, ranging from identity theft to personal data leakage. According to the IBM report, the global average data breach cost in 2023 was USD 4.45 million, which is a 15% increase over 3 years [1]. As a consequence, securing these organizations is crucial to maintaining society’s functioning.

Cyber security is a significant practice for protecting networked systems and information from unauthorized access, disclosure, or modification. Traditional techniques, including antivirus and firewalls, may not be effective due to the large implementation of booming technologies such as the Internet of Things (IoT).

Artificial intelligence (AI) is rapidly transforming the cyber security landscape. Several organizations adopt AI-powered cyber-security solutions to detect and respond to threats more effectively and efficiently than ever before. Machine learning (ML), a subset of AI, is a promising technique that provides automation and intelligence to tackle various cyberattacks [2]. The convergence of ML and IoT significantly enhances the capabilities of IoT-based security systems, making them capable of handling sophisticated security threats. For example, ML algorithms can analyze data collected by IoT devices to detect unusual patterns that may indicate a cyberattack or intrusion. On the other hand, ML systems are being exposed to several serious security vulnerabilities. An adversary, for example, could manipulate training data to control and degrade learning models’ expected behaviors. This field is known as adversarial machine learning (AML), which focuses on examining attacks designed to undermine the performance of ML-based models. It also explores creating and identifying adversarial examples and how to integrate potential defensive strategies.

AML has attracted remarkable interest in recent years. Researchers in [3] investigated the application of AML attacks and defenses in the context of IoT specifically in ML-based network intrusion detection systems. In [4], the authors reported AML techniques for IoT-enabled 5G networks. The study [5] deals with attack techniques against IoT-based ML systems, where the authors shed light on the severity of such vulnerabilities and the necessity of protecting ML models from rising malicious attacks.

Although several studies have identified the potential susceptibility of ML models to adversarial attacks, the AML field is still growing. The impact of AML in IoT-enabled security systems remains insufficiently explored. Moreover, the critical importance of examining the impact of AML attacks and defenses is still needed, since many IoT systems are part of critical infrastructure (e.g., smart healthcare), where security breaches can have severe consequences. Therefore, studying AML in the context of IoT helps with identifying and mitigating the risks posed by adversarial attacks and ensuring the integrity and safety of IoT applications. It also contributes to the advancement of security practices and technologies in this rapidly evolving field. This paper addresses these gaps by presenting a taxonomy of AML and providing an up-to-date systematic review of AML in IoT-based security systems. It will assist readers by delving into the evolving field of cyber security leveraging AML and IoT technologies, aiming to develop resilient, secure, and reliable IoT systems. The key contributions are summarized as follows:

- A comprehensive taxonomy of AML is provided.
- A systematic review of relevant studies that tackle the AML in IoT security systems.
- A comparison of recent related studies based on notable features.
- A roadmap of future directions motivated by the unsolved issues.

**Figure 1:**
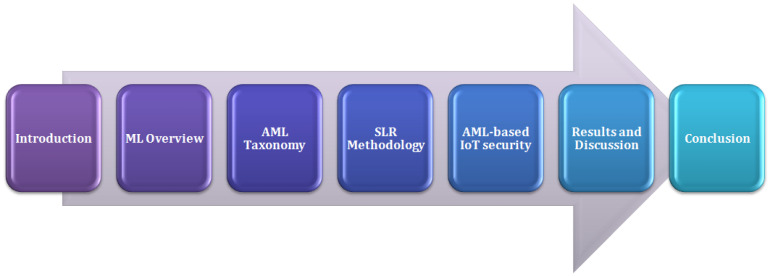
Paper structure.

The paper structure is depicted in Figure 1. Section 2 introduces the fundamental concepts of ML. A comprehensive taxonomy of AML is proposed in Section 3. In Section 4, we present the systematic literature review process to address the AML challenge in the IoT context. In Section 5, we analyze the selected research articles. The main findings of this study are discussed in Section 6 and conclusions are provided in Section 7.

## 2. Machine Learning Overview

We introduce the fundamentals of ML in order to pave the way for the audience to understand the context of AML. It should be noted that this is not an exhaustive introduction and that interested readers can refer to references [6,7].

ML is a powerful AI tool that can be used to solve a wide variety of problems. It is generally classified into supervised, unsupervised, and reinforcement learning. Supervised learning uses labeled data in training models to yield the desired output. The algorithm learns to predict the output for new inputs based on the patterns learned from the training data. It can be separated into classification and regression. Support vector machine (SVM), decision tree (DT), k-nearest neighbor (KNN), and random forest (RF) are common supervised ML algorithms. In contrast, unsupervised learning is defined by its ability to group unlabeled data based on their similarities or differences. It determines hidden patterns from the dataset and can be used in clustering, association, and reduction tasks. In reinforcement learning, an intelligent agent learns the optimal behavior in a specific environment to achieve the most suitable actions. Unlike supervised and unsupervised learning models, the input data are accumulated from the environment. The reinforcement algorithms aim to obtain a maximum reward for the agent. Policy-based, value-based, and model-based mechanisms can be used to implement reinforcement learning.

Recently, self-supervised learning has emerged as a nascent paradigm that is expected to improve several complex tasks, such as natural language processing (NLP). The model is trained using unlabeled data on the first iteration and then generates data labels automatically by identifying useful features in the input data. This process is known as pretext learning and is based on several tasks, such as color and geometric transformation.

**Figure 2:**
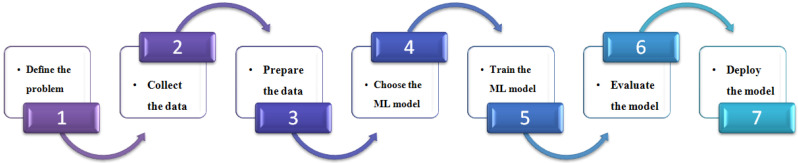
Machine learning workflow.

Figure 2 depicts the ML workflow, which consists of a series of steps to develop and deploy an ML model. First, it involves defining the problem by identifying the issue to solve and the type of predictions the model will make. Next, it involves collecting data relevant to the problem being solved, as the quality of the dataset significantly impacts the model’s performance. After collecting the data, it needs to be prepared for training. This process might include cleaning the data, eliminating outliers, and converting it into a format that is compatible with the ML algorithm. There are many different ML algorithms available, each with its strengths and weaknesses. It is important to choose an appropriate algorithm for the problem and data. After selecting the algorithm, the model is trained using the prepared data. This process entails providing the data to the algorithm and enabling it to learn the patterns within the data. Once training is complete, the model’s performance is assessed using a separate test set, which helps determine how effectively the model will generalize to new data. Once the model achieves the required performance, it is deployed to production. This may involve integrating the model into a software application or making it available as a web service.

## 3. Adversarial Machine Learning

**Figure 3:**
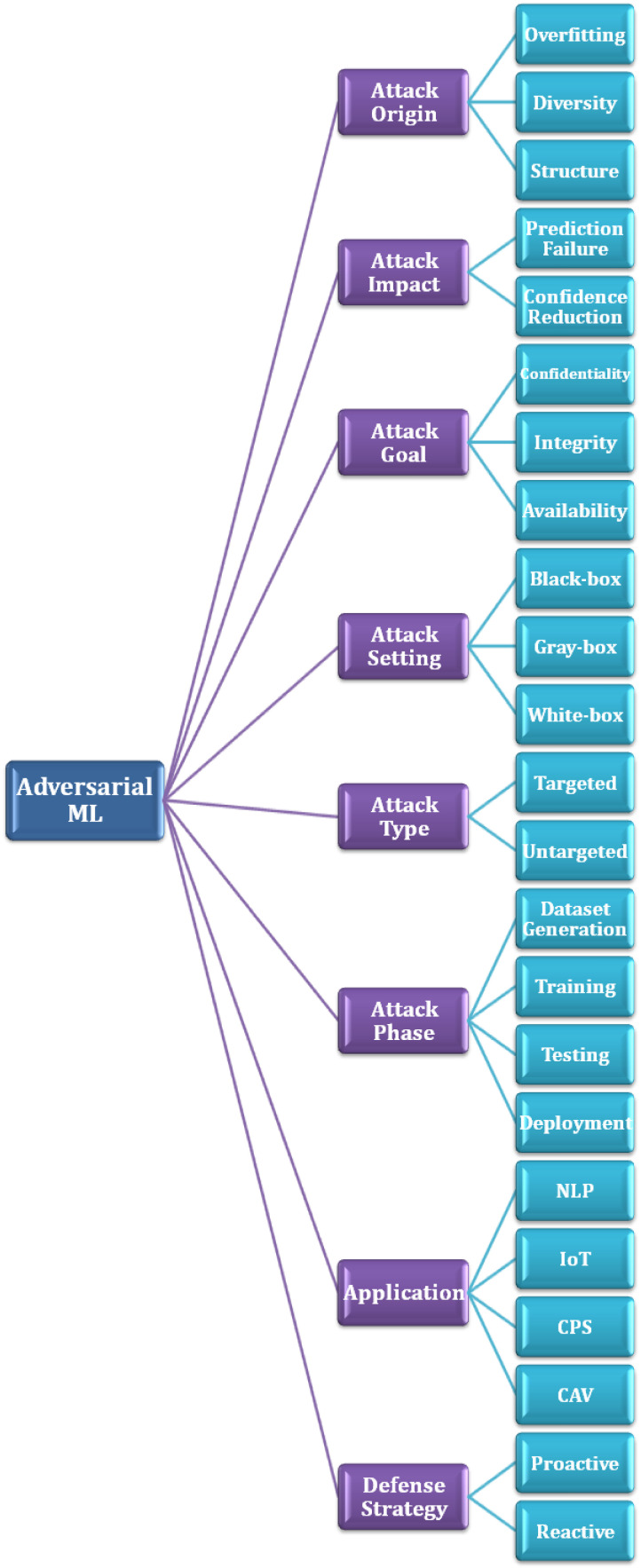
Adversarial machine learning.

AML is a field of study that focuses on designing attacks to compromise the security of ML models and developing defenses against these threats. Figure 3 illustrates a taxonomy of AML including origins, impacts, goals, setting, and phases of attacks. It also shows the commonly targeted applications and main defense strategies that can be used to protect against adversarial attacks. Table 1 provides a short description of each attack.

### 3.1. Attack Origins

Overfitting is an instrumental factor allowing adversarial attacks that is caused by the complexity of the ML model and the limited size of the training data. ML models are usually trained using hundreds of epochs, which renders the memorization of training instances difficult. Moreover, a target model is overfitted when a difference in training accuracy and test accuracy occurs. This can elevate the attack success rate [8].

Diversity also plays a crucial role; an ML model performs better when it is trained with a large number of records (i.e., it will be less prone to overfitting). However, the diversity of training data could increase privacy risks, especially in self-supervised learning models.

Additionally, the structure of ML models influences their behavior and predictions on the given training data. Consequently, some ML models are more prone to adversarial attacks. For example, the robustness of the support vector machine, stochastic gradient descent, logistic regression, random forest, Gaussian naive Bayes, and k-nearest neighbor algorithms is evaluated under data-poisoning attacks using four datasets in [9].

### 3.2. Attack Impacts

Prediction failure refers to the effect caused by successful adversarial attacks in increasing misclassification errors and rendering the target system ineffective (i.e., availability violation).

Moreover, confidence reduction occurs when an adversary manipulates the training dataset to corrupt the security of the target system and degrade its confidence scores.

### 3.3. Attack Goals

Confidentiality and privacy are threatened if an attacker can obtain confidential and private information by querying the ML system, such as accessing the training dataset or stealing the classifier parameters. Integrity is compromised where an attacker can modify feature vectors and labels or add noisy data to cause the misclassification of the ML classifier. Availability issues arise where an attacker can generate malicious sessions, causing the system to classify the traffic as malicious to block legitimate traffic and make the ML system unavailable.

### 3.4. Attack Setting

We can classify adversarial attacks into three main categories based on the adversary’s knowledge about the targeted ML model. In black-box attacks, the adversary does not know the ML model and queries it as a black box. The adversary has a limited degree of knowledge about the targeted model in gray-box settings, such as the confidence score provided by the classifier. While the adversary knows the model architecture and hyperparameters used for the model training in white-box settings.

### 3.5. Attack Type

Targeted attacks occur when an adversary tries to manipulate specific input data to force the ML model to produce an intended prediction.

On the other hand, untargeted attacks involve an adversary tampering with any class of data to deteriorate the performance of the ML model and reduce the classification confidence.

### 3.6. Attack Phase

Dataset generation is the process of gathering and collecting information from different sources. This dataset is used to train the ML model. It is recommended to collect a large amount of data to provide efficient ML models. An attacker can access the dataset and change the values of randomly selected features, resulting in a noisy dataset. Moreover, the attacker may have prior knowledge of the statistical distributions of different features and generate a synthetic dataset [10]. Some ML applications require private individuals’ data that will be uploaded to centralized servers in plaintext to extract patterns and build ML models, causing serious data privacy leakage.

**Figure 4:**
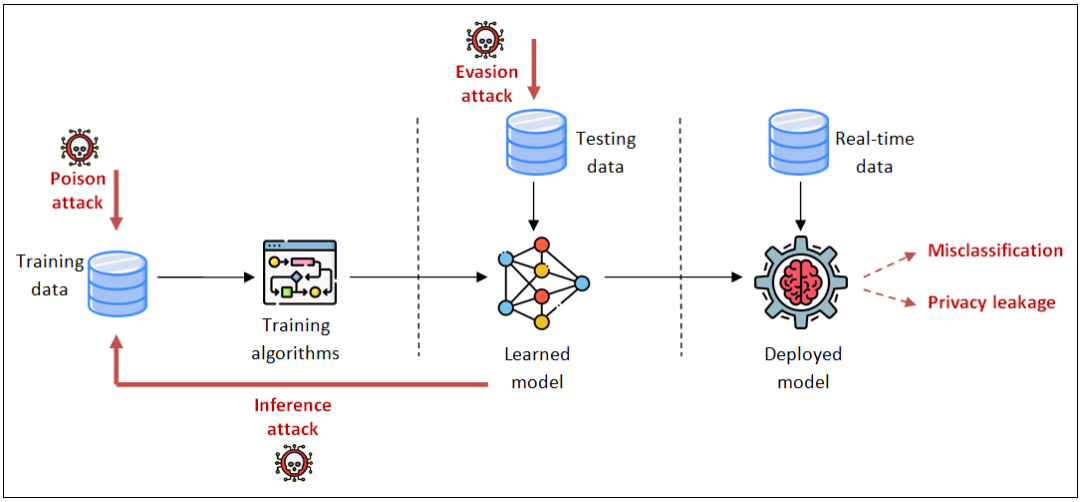
Machine learning major attacks.

The training phase is one of the fundamental steps in developing ML-based systems. In this phase, an algorithm learns from the generated dataset to develop ML solutions. In poisoning attacks, the attacker begins with a new training dataset and manipulates it to create a different dataset, which is then used to train the learning algorithm (see Figure 4). In contrast, backdoor attacks allow the adversary to insert poison samples containing the backdoor trigger into the dataset (i.e., a pattern that is smoothly introduced to any input) [11]. The injected backdoor forces the model to produce unexpected behavior.

The testing phase is the process of evaluating the trained model with a testing dataset that is a part of the same training dataset. This phase is carried out after the model training phase and aims to test the generalization ability of a trained model. However, a malicious adversary can exploit the vulnerabilities of the ML-based system and modify only samples from the test set to evade detection by the model. The most common types of attacks launched during this phase are evasion, impersonation, and inversion attacks [12].

Prediction or deployment is the task of integrating an ML model into the deployment environment to provide decision making by exploring data and identifying patterns with minimal human intervention. Some real-world datasets may contain sensitive information; an adversary can use the confidence score of the ML-based model to reconstruct additional features of the same set of samples and cause severe privacy leakage [13]. Potential attacks such as data manipulation and model stealing can occur in the deployment phase. The end goal of such attacks is to reveal sensitive information, make incorrect predictions, or corrupt the ML models.

### 3.7. Applications

AML applications span a range of areas, focusing on attacking ML systems in key applications.

Natural Language Processing (NLP) involves the treatment of human language based on ML to provide prevailing tasks such as sentiment analysis, text classification, and smart assistants. Although ML has revolutionized the field of NLP, it is not resilient against adversarial attacks that introduce perturbations at different levels, including character, word, or sentence levels. Common techniques can be utilized by adversaries, such as insertion, flipping, and deletion, to undermine the model outputs [14].

Internet of Things Systems (IoT) are growing in prominence, ranging from consumer-oriented to enterprise-oriented applications where ML models have been adopted in a plethora of IoT systems, specifically those that need a decision-making process. However, in the context of IoT, ML models are not truly trained in a benign environment, thus introducing adversarial threats during the training and testing phases. Prevalent techniques such as the Gradient Ascent Method (GAM), Fast Gradient Sign Method (FGSM), Projected Gradient Descent (PGD), and Carlini and Wagner Method (C&W) can be exploited and launched by malicious opponents to subvert and degrade the performance of ML-based IoT systems [15].

Cyber Physical Systems (CPSs) seamlessly integrate computational entities with the physical world through the Internet to provide potential services without human implication. In an ML-based CPS, the data used for training and testing purposes are usually exchanged via open channels, leading to various adversarial attacks such as data poisoning, model poisoning, privacy breach, and runtime disruption [16]. As a result, the ML model will produce unexpected outcomes.

Connected and Autonomous Vehicles (CAVs) are an emerging area of research that is expected to provide an impressive effect on the future vehicular ecosystem due to automation and cooperation capabilities. ML has a paramount role in building CAV systems, including perception, prediction, planning, and decision making. However, poisoning or evasion attacks can be launched to compromise the training or inference phase, respectively. In addition, different types of perturbations can be generated to make the system produce the intended results [17].

### 3.8. Defenses

In a reactive strategy, the target system reacts to adversarial attacks as they arise. For instance, applying machine unlearning after data poisoning attacks involves an unlearning algorithm to remove the influence of poisoned data from the training dataset and the trained model. As a result, the model performance and resilience will increase.

In contrast, a proactive strategy involves anticipating and preparing for potential adversarial attacks before they happen. Most defense methods are proactive, where the target system anticipates suitable solutions before the occurrence of adversarial attacks to handle damages as much as possible. For example, we first train a machine learning model on a dataset of both normal and adversarial examples. This helps the model learn to be more robust to adversarial attacks. Furthermore, we check the input data for suspicious patterns that may indicate an adversarial attack. If suspicious patterns are found, the input data can be rejected or sanitized.

## 4. Review Methodology

**Figure 5:**
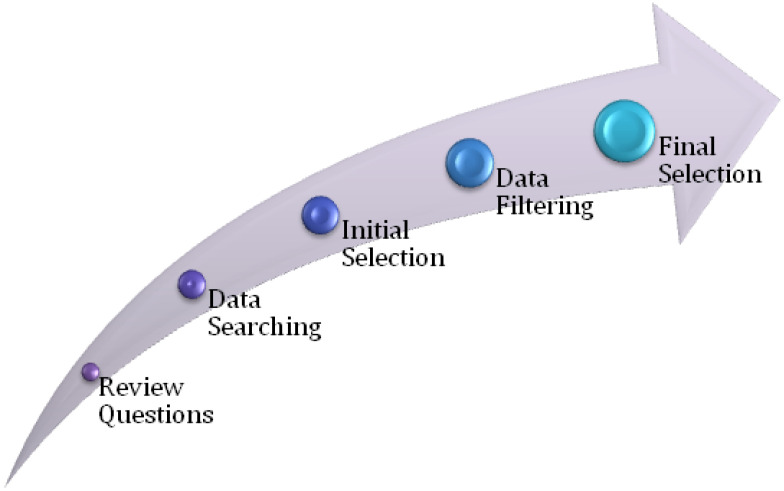
Systematic review process.

This work explores how adversarial machine learning affects IoT systems using the systematic literature review (SLR) approach. Figure 5 outlines the steps taken in this study. Our review process involves five key stages: formulating review questions, conducting data searches, initial selection, data filtering, and final selection.

**Research Questions:** First, we defined the following review questions (RQs):

- **RQ1**: How do AML attacks influence the functionality of IoT-enabled security systems?
- **RQ2**: Which AML defense strategies are recommended to counteract these attacks?
- **RQ3**: What are the open issues and future directions related to AML in the context of IoT?

**Data Searching:** Second, we identified relevant keywords for conducting searches on Google Scholar, which is a widely used electronic database comprising reputable scientific publishers like IEEE, Elsevier, ACM, and MDPI, among others. Employing Boolean operations, we utilized <AND> to pinpoint primary search terms and <OR> to include synonyms and alternate spellings of these main keywords.

- “Adversarial Machine Learning” <OR> “AML”

<AND>

- “Internet of Things” <OR> “IoT”

<AND>

- “attacks” <OR> “defense”

**Figure 6:**
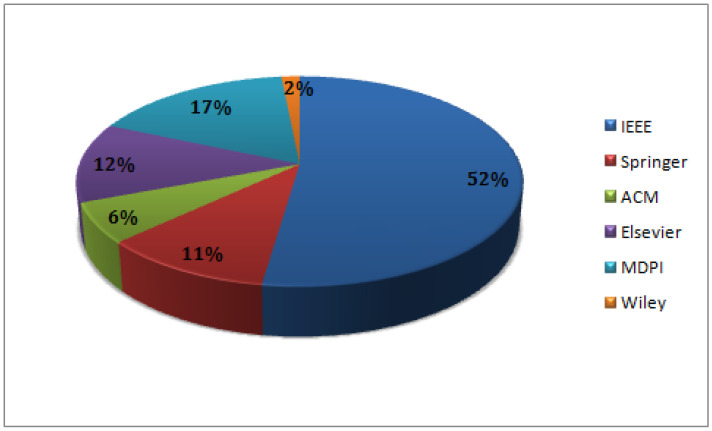
Distribution of initial selected papers among publishers.

**Initial Selection:** Next, we selected 65 papers released within the past five years (from 2020 to 2024). Figure 6 illustrates the distribution of research publications among various publishers. The majority of research papers are found in five prominent databases: IEEE, Elsevier, ACM, MDPI, Springer, and Wiley, accounting for 52%, 12%, 6%, 17%, 11%, and 2%, respectively. However, an adversarial example creation of a dataset with balanced classes is still difficult to implement in a real-world setting.

**Data Filtering:** Following the initial selection of several published papers by analyzing the titles and abstracts of each article, we applied the inclusion and exclusion criteria outlined in Table 2.

**Figure 7:**
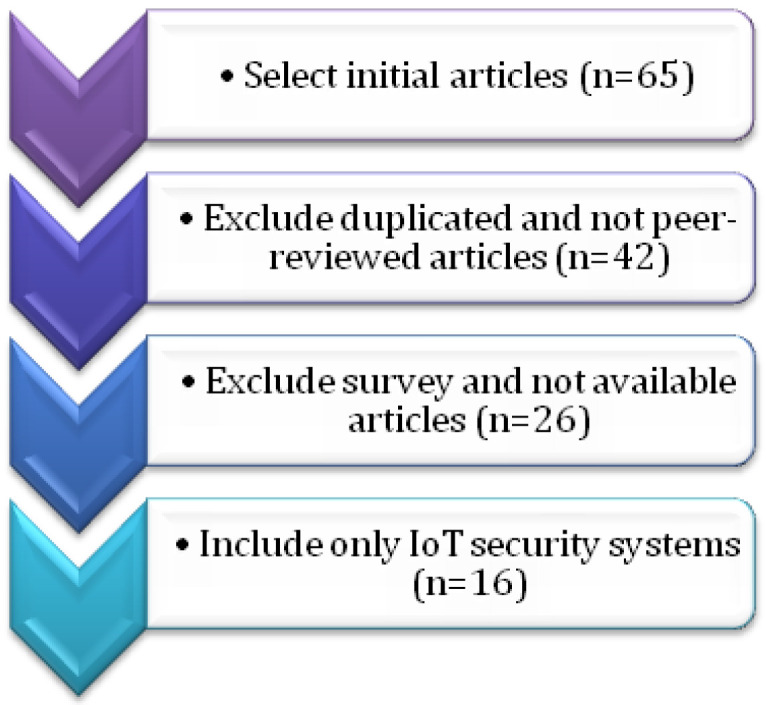
Selection of final articles.

**Final Selection:** Finally, a total of 16 research articles were chosen to address the identified analytical queries within the systematic review. Figure 7 depicts the process of final study selection, which is guided by the principles of inclusion and exclusion.

## 5. AML-Based IoT-Enabled Security Systems

In this section, we critically describe the final selected articles to provide a thorough summary of the current literature, with the aim of answering the questions under consideration. We also provide a comparison of the relevant studies in Table 3.

Dunn et al. [18] explored the effects of data-poisoning attacks on machine learning models such as gradient boosting machine (GBM), random forest (RF), naive Bayes (NB), and feed-forward deep learning (FDL) within IoT networks. Initially, all models were trained on the original ToN_IoT and UNSW NB-15 datasets in a non-adversarial setting. Subsequently, a label modification function was used to introduce poisoned data at different rates by altering the normal classes in both datasets. Label modification is a method used when an adversary cannot inject new inputs but can change the labels of existing data in supervised learning datasets. The models were evaluated based on accuracy, precision, false positive rate, and detection rate. The findings indicated that when the amount of normal data is not significantly greater than the poisoned data, accuracy decreases and false positive rates increase.

Qiu et al. [19] studied the impact of adversarial attacks on the deep neural network (DNN) model, specifically on the DNN-based network intrusion detection system (NIDS) for IoT. They presented two steps: model extraction, which involves replicating the deep learning model using a limited amount of training data, and a saliency map, which identifies key features that influence detection outcomes. Subsequently, they applied the Fast Gradient Sign Method (FGSM) to create the desired perturbations. They applied their method to attack *Kitsune*, which is a cutting-edge NIDS that identifies anomalies in IoT through traffic analysis. They assumed that the adversary is aware of the mechanism used in this NIDS system, as it is typically public knowledge, but it does not have access to the specific parameters and hyperparameters of the deep learning model. In addition, the adversary is capable of installing a malicious network device within the same network to passively monitor traffic flows and actively alter traffic characteristics. The model extraction phase involves reconstructing a shadow model that mimics the behavior of the target model. The adversarial example (AE) generation phase utilizes the saliency map to pinpoint the crucial elements in the traffic features that influence detection results. It then employs a gradient-based optimization method (FGSM) to create AEs by altering these key elements. The proposed attack method significantly raises the number of false positives and false negatives in scenarios involving the Mirai dataset and VideoInjection dataset. However, the authors did not address mitigation strategies to improve the robustness of DNN-based IoT intrusion detection.

Vitorino et al. [20] examined the robustness of tree-based algorithms and ensembles in detecting intrusions in IoT networks. The study introduced an evasion attack method aimed at three supervised algorithms: random forest (RF), extreme gradient boosting (XGB), and light gradient boosting machine (LGBM), as well as an unsupervised algorithm: isolation forest (IFOR). The classification assumed the attacker had no access to the model’s training set or internal parameters. Two scenarios for IoT network intrusion detection were considered: binary and multiclass classification, using the IoT-23 and Bot-IoT datasets. The evasion attack employed the adaptive perturbation pattern method (A2PM) on both regular and adversarial training. While the accuracy of all models decreased with regular training under attack, they retained high accuracy with adversarial training.

Taheri et al. [21] proposed two defense strategies to enhance the Android malware detection system in IoT environments, specifically targeting the adversarial FGSM attack on the SVM classification model. Both strategies utilize the nearest neighbor concept to rectify the perturbed training set. The first strategy, termed robust nearest neighbor (Robust NN), increases the number of neighbors used to label a sample when the nearest neighbor does not match the sample. The second strategy, a combination of a convolutional neural network and 1-nearest neighbor (C4N), integrates 1-NN and CNN classification methods to train another CNN classifier, which then mitigates the adversarial samples in the training set. The effectiveness of these methods was assessed using three Android malware datasets: Drebin, Genome, and Contagio. The results indicated that both defense methods achieved high accuracy but required significant processing time when handling large datasets.

In another work, Taheri et al. [22] targeted Android malware detection in industrial IoT (IIoT) systems. They introduced a federated learning-based architecture comprising client and server sides. On the client side, various participants, including potential adversaries, can locally train the model using generated poison data. This undermines the accuracy of the detection system and enables the insertion of more malware samples into the system. The attacker employs two generative adversarial network (GAN)-based algorithms to create adversarial examples and inject them into the datasets of each IIoT application. The server side implements a defense mechanism that detects abnormal behaviors by clients, avoiding anomaly aggregation through a GAN network called A3GAN. This mechanism accurately identifies malicious models (based on a threshold) and eliminates poisoned samples during the aggregation process. The authors tested their attack and defense methods using three Android malware datasets: Drebin, Genome, and Contagio. Both methods demonstrated high performance. However, the security of data transmitted between participants and the server was not addressed, leaving the system vulnerable to inference attacks that could extract information from the exchanged model updates.

Ding et al. [23] addressed the issue of preserving the privacy of images stored on electronic IoT devices against DNN-based privacy leakage by using adversarial examples, specifically by replacing photos stored on mobile phones with these examples. Their work concentrated on creating adversarial examples that are imperceptible to the human eye while being computationally efficient. They employed the selective gradient sign iterative method (SGSIM) and momentum selective gradient sign iterative method (M-SGSIM). These methods minimize image distortions in adversarial examples by ignoring pixels with low partial derivatives during the iteration process. The authors validated their approach through non-targeted and targeted adversarial attacks, demonstrating that their methods can effectively mislead the Inception v3 classifier, a deep convolutional neural network model, with minimal perturbations and computational cost. However, they did not explore the impact of these adversarial examples on other ML models.

Mamunur et al. [24] evaluated the susceptibility of existing ML-based IDS models against adversarial attacks in the context of IoT networks used in smart city applications. They also suggested hardening the models by retraining them using carefully constructed adversarial samples. Five adversarial attack techniques have been applied in the work to produce adversarial samples, and they have generated adversarial samples using the deep neural network (DNN) model, which uses test data as input. The authors explored the impact of five adversarial attacks, namely Jacobian-based Saliency Map Attack (JSMA), FGSM, Basic Interaction Method (BIM), Carlini and Wagner (CW) attack method, and Deep Fool (DF), on ML-based IDS models for detecting intrusion within an IoT network. The results showed that all ML models were vulnerable to adversarial attacks, and retraining the model with a portion of the generated adversarial samples improved performance. The results indicated that the CW method had the greatest adverse impact on the models’ performance, generating extremely low scores across all frequently employed metrics of performance. However, the authors did not discuss how these models might be designed to be less vulnerable to adversarial attacks.

Pavlos et al. [25] analyzed the Bot-IoT dataset to assess robustness against adversarial examples. They conducted label noise adversarial attacks on an SVM model and created adversarial examples using the FGSM against both binary and multiclass Artificial Neural Network (ANN) models. First, they replicated the linear SVM model to accurately evaluate metrics like accuracy, recall, precision, and F1-scores. An ANN was employed for training and evaluating data more quickly than Recurrent Neural Networks (RNNs) and Long Short-Term Memory (LSTM) models. Despite this, the activation functions and neural network structure remained fixed for precise comparison. The subsequent stages focused on adversarial cases for the SVM and ANN. During training, they generated noise using SVM adversarial examples. They thoroughly evaluated the experiment’s outcomes and the model’s resilience to adversarial instances. Their findings revealed that Bot-IoT classes are imbalanced, which they addressed by adjusting the models’ class weighting parameters. However, creating adversarial examples in a dataset with balanced classes in a real-world setting remains challenging.

The authors in [26] investigated the impact of adversarial attacks on network security, focusing on the vulnerability of Intrusion Detection Systems (IDSs) to adversarial examples. By employing the FGSM to generate adversarial examples, the robustness of IDS models based on CNN, LSTM, and gated recurrent unit (GRU) is evaluated. Three training methods are compared: training with normal examples, training with adversarial examples, and a combination of pretraining with normal examples followed by adversarial training. The results show that CNN is the most robust model under normal training, while LSTM and GRU exhibit improved robustness after adversarial training. Enhancing the resilience of IDS against adversarial attacks is crucial for network security in the context of IoT. However, adversarial examples crafted to exploit vulnerabilities in deep learning models can lead to false positives or false negatives, compromising the reliability and effectiveness of intrusion detection systems.

Jiang et al. [27] introduced a novel model, Feature Grouping and Multi-model fusion Detector (FGMD), which effectively defends against adversarial attacks on IoT network intrusion detectors. By utilizing feature grouping and multi-model fusion, the FGMD enhances detection accuracy and resilience against attacks. Experimental results demonstrated the model’s effectiveness in detecting adversarial samples while maintaining performance in the absence of such attacks. The authors provided a valuable defense mechanism against adversarial threats. However, deep learning-based intrusion detection schemes typically require a large amount of labeled data for training.

Vitorino et al. [28] introduced the adaptative perturbation pattern method (A2PM) to address adversarial attacks in machine learning, particularly in the IoT security domain. A2PM focused on generating realistic adversarial examples that can evade detection with a specific emphasis on tabular data domains. The proposed method used separately customized pattern sequences to generate valid and coherent data perturbations tuned to particular class features. A2PM was evaluated against multilayer perceptron (MLP) and random forest (RF) classifiers using the CIC-IDS2017 and IoT-23 datasets. The results demonstrated the scalability and effectiveness of A2PM in generating realistic adversarial examples, demonstrating potential advantages for adversarial training and discovering weaknesses in machine learning models. However, deploying adaptive perturbation patterns in large-scale network environments with numerous devices and heterogeneous architectures may pose scalability challenges.

The authors in [29] proposed a solution aiming to enhance security defense against adversarial text-based CAPTCHA attacks in IoT devices connected to web services. The adversarial training is performed using three main attack methods: the FGSM, the Iterative Fast Gradient Sign Method (I-FGSM), and the Momentum Iterative Fast Gradient Sign Method (MI-FGSM) algorithms. Meanwhile, the defense model uses a CNN with Denoising Autoencoder (DAE-CNN) as the primary machine learning model to break adversarial attacks. The author proposed including the introduction of a Mixed Batch Adversarial Generation Process (MBAGP) to enhance the robustness and computational efficiency in training the model with both original and adversarial CAPTCHA images. However, the high computational complexity required due to the generation of adversarial images during each batch update may lead to higher processing demands.

Lurski et al. [30] addressed the vulnerability of DL-based IDSs to data-driven adversarial attacks applied in the context of wireless sensor network routing in IoT. Attackers strategically adjust their behavior to degrade the DL-based IDS model and evade the detection of conventional attacks. The attack setting involves the application of an oracle evasion attack (OEA) in which attackers with access to the model will be able to evaluate how their current features are classified through either a stolen IDS model from a compromised node or inferred via an exploratory attack. The attackers then adjust their behavior accordingly by directly modifying controllable features (perturbation vector), such as the Received Signal Strength Indicator (RSSI), so that the IDS would classify them as benign instead of the true label of the attacker. Therefore, the authors proposed a defense technique that combines both adversarial example training and outlier detection. The adversarial example training involves the training of the IDS model on generated perturbations using a modified FGSM that takes into account variable scalar and different weighted features. The outlier detection aims to identify samples with uncharacteristically high perturbation scalars that could lead to misclassification. Using a public available dataset (WSN-DS), the authors demonstrate the effectiveness of combining adversarial example training and outlier detection strategies in diminishing the potential attack space. However, this strategy involves creating a separate model to identify outlier behavior and distinguish between legitimate data and adversarial examples.

Anthi et al. [31] investigated the behavior of a range of supervised classification algorithms used for IDSs in IoT networks against AML attacks. They used two relevant techniques, including the FGSM and JSMA, to automatically generate perturbed samples. The adversarial samples were subsequently included along with the benign testing data points and presented to the DT trained model. This latter has a high decrease in precision, recall, and F1-score. Moreover, they included adversarial training to defend against such attacks and provide better performance.

Pantelakis et al. [32] proposed an ML-based multiclass classification methodology for cyber-attack detection in IoT networks. They investigated the robustness of RF, DT, KNN, and MLP classifiers against JSMA, the FGSM, and DeepFool adversarial attacks which target the testing phase of ML-based IDSs having access to the training data. To this end, they deployed an adversarial training in order to experiment with a large dataset created from the traffic that contains multiple attacks, such as DoS, Scan, MiTM and Mirai in the activities of a smart home IoT network. The authors demonstrated that adversarial training can overall enhance the classifier’s robustness without affecting the performance on normal samples.

Alkadi et al. [33] developed a robust ensemble adversarial machine learning framework (RobEns) aiming to enhance the performance of IDS in IoT environments. They used ensemble methods to increase the complexity for adversaries, thus improving the robustness of IDSs and ensuring a comprehensive defense mechanism against adversarial attacks, including the FGSM, C&W, Zeroth Order Optimization (ZOO), and HopSkipJump attacks. The defense methodology employed in the proposed framework is based on a proactive association of data and model-based modifications, forming an ensemble-based defense technique with two key components/modules. The feature squeezing module reduces the feature space to limit potential perturbations by adversaries. The adversarial training aims to enhance model robustness by training on adversarial examples. The experiments’ validation using three benchmark datasets demonstrates that the proposed framework enhances the robustness and improves the detection rates of IDS models in the IoT context. However, this framework requires continuous updates and fine tuning to adapt to evolving attack methods.

## 6. Results and Discussion

In this section, we conduct a statistical analysis of the SLR results and address the analytical questions **RQ1**, **RQ2**, and **RQ3**.

**Figure 8:**
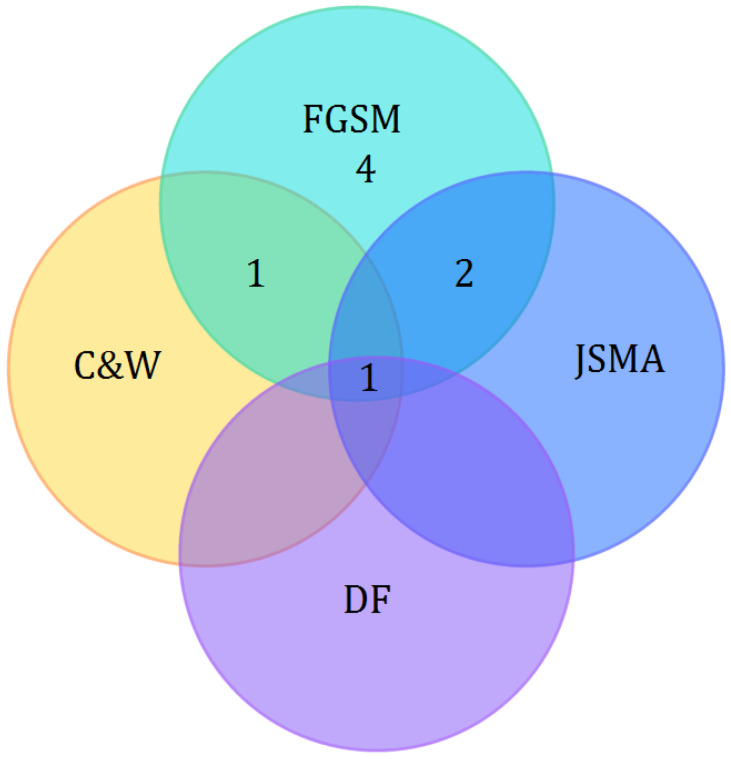
Common attack methods with research studies.

- **RQ1**: How do AML attacks influence the functionality of IoT-enabled security systems? AML attacks can significantly impact the functionality of IoT-enabled security systems by exploiting vulnerabilities in the ML models. In poisoning attacks, the compromised model might learn incorrect patterns, leading to increased false positives or false negatives in threat detection. For instance, an IoT-based IDS might be trained to ignore malicious network traffic or suspicious activities that may lead to unauthorized access attempts, malware propagation, or data breaches. These IoT-enabled intrusion detection systems are crucial in protecting critical infrastructure sectors such as energy, healthcare, and transportation by monitoring for unusual activities that could indicate a cyberattack aimed at disrupting essential services. In evasion attacks, adversaries craft input perturbations that are intentionally designed to be misclassified by the ML model. Consequently, IoT-enabled security systems fail to detect malicious activities. Moreover, adversaries could replicate the ML model by querying it and analyzing the outputs, leading to undermining the security and privacy of IoT ecosystems.
  Figure 8 depicts the most used attack methods in IoT-enabled security systems. Gradient sign-based methods such as the FGSM are the most employed, which leverage the gradients of the loss function with respect to the input data to create adversarial examples. The basic idea is to make a small perturbation to the input data in the direction that maximally increases the loss. This perturbation is designed to be imperceptible to humans but sufficient to fool the ML model. In critical infrastructure systems, adversarial attacks using the FGSM can cause operational disruptions. For instance, an adversarial perturbation could fool an IoT-based industrial control system into misinterpreting sensor data, leading to improper control actions. There exist other methods such as DF, JSMA, and C&W that can significantly reduce the accuracy of IoT security systems. Unlike the simple FGSM method, DF is an iterative and more refined approach that seeks to find the smallest perturbation necessary to misclassify an input. The JSMA is a targeted adversarial attack method that creates adversarial examples by leveraging the Jacobian matrix of a neural network’s predictions with respect to its inputs. The C&W attack, proposed in [34], is known for its ability to craft adversarial examples with minimal perturbations that are difficult to detect and highly effective in fooling deep neural networks.
- **RQ2**: Which AML defense strategies are recommended to counteract these attacks? To mitigate the risks posed by AML attacks on IoT-enabled security systems, several strategies can be employed. According to studies [20,24,25,26,29,30,31,32,33], adversarial training is a powerful technique to enhance the robustness of IoT-enabled systems against AML attacks. It involves incorporating adversarial examples into the training process of an ML model by combining the original training data with the generated adversarial examples. Therefore, the model trained with adversarial examples is better at resisting AML attacks, as it learns to correctly classify both clean and perturbed inputs. However, the process of adversarial training can be time consuming due to the generation of adversarial examples and dataset augmentation.
- **RQ3**: What are the open issues and future directions related to AML in the context of IoT? The rapid expansion of ML-based models to embellish the design of various systems has been followed by numerous relevant threats. These models attempt to guarantee accurate and efficient predictions and outcomes. However, these goals are encountered by corresponding vulnerabilities that jeopardize and degrade the performance of ML models. Therefore, it is crucial to employ defense mechanisms at different phases of the model construction. Furthermore, we identify the following future directions that could be addressed by interested researchers.
  - In fact, most ML models require a large set of data gathered from a variety of sources of IoT devices during the training phase, which can reveal the private information of users. Although federated learning (FL) was introduced to protect data privacy by sharing only local model updates from multiple users, it is still challenging to ensure the impact of these local updates on the global model performance. In addition, in homogeneous FL, each local party is assumed to have the same model architecture. This is not true in real-world IoT applications since each party’s capacity for computation and communication may differ. Hence, future efforts are required to design privacy-preserving FL models in a heterogeneous context.
  - In poisoning and backdoor attacks, the adversary can inject poison examples into the training dataset to significantly change the model predictions. Hence, access control mechanisms, outlier-based techniques, and anomaly detection are required during the model training.
  - Generating additional tasks to provide a clean dataset and safeguard the ML-based IoT model integrity is computationally expensive and could not be suitable for real-world applications. Therefore, designing a secure ML model with lightweight computation is needed for practical IoT applications.
  - In the white-box setting, the adversary has knowledge of hyperparameters and thus could select the attack methods accordingly. Several studies tackle adversarial attacks on ML-based IoT systems. However, it is still challenging to develop defense methods that are robust against white-box attacks. Specifically, designing effective methods to mitigate all types of attacks and not only a specific one is also difficult. In addition, some standard criteria that need to be introduced and followed to evaluate the resilience of ML models against adversarial attacks make implementing defense mechanisms more challenging.
  - Machine unlearning is a nascent ML field that requires removing samples from the trained model. It can be efficiently employed as a post-attack solution since predicting all possible attacks throughout the ML lifecycle is not feasible in real-world IoT applications. However, the unlearning operation jeopardizes the privacy of detailed information about the unlearned samples. Therefore, it is crucial to explore data privacy during the unlearning process.

## 7. Conclusions

AML in IoT is a critical area of concern given the increasing reliance on IoT systems in various sectors. In this paper, we investigated the AML field in IoT-enabled security systems using the systematic review methodology. We presented an overarching taxonomy of AML including attack origins, impacts, goals, settings, types, phases, applications, and defense strategies. We provided an up-to-date systematic review of AML in IoT-based security systems to determine how AML attacks can influence the functionality and degrade the performance of such systems. The statistical analysis revealed that the FGSM is the most widely used attack method. The FGSM alone or in conjunction with other methods such as JSMA can notably reduce the accuracy of IoT security systems. The exploration of adversarial attacks draws attention to the vulnerabilities of ML models, inciting the development of defense strategies. According to the reviewed recent studies, implementing robust defense strategies such as adversarial training (i.e., training ML models with adversarial examples) can significantly enhance the effectiveness of IoT systems.

As IoT continues to expand and evolve, addressing the challenges posed by adversarial ML will be critical for ensuring the security, reliability, and trustworthiness of connected devices and systems. The future of AML in IoT involves high-quality datasets, adversarial training, real-world testing, and continuous monitoring.

## Figures and Tables

**Table 1:** Brief description of potential ML attacks.

Security Attack	Description
Poisoning attack	An attacker manipulates the training data to mislead the learning of the ML model
Backdoor attack	An attacker injects a small pattern into the ML model during training to provide misbehavior
Evasion attack	An attacker alters the test data to undermine the integrity of the ML model
Impersonate attack	An attacker steals the data record to act as the targeted ML model
Inversion attack	An attacker creates feature vectors that resemble those used to create an ML model
Reconstruction attack	An attacker reconstructs the raw data using its white-box knowledge of the feature vectors
Membership inference attack	An attacker determines if the sample was a member of the training set used to build the ML model

**Table 2:** Inclusion and exclusion criteria of our work.

Inclusion Criteria	Exclusion Criteria
- Articles published between 2020 and 2024	- Duplicated and not peer-reviewed articles
- Articles published in journals/conference	- Survey and non-available articles
- Articles that address IoT security systems	- Articles that address other IoT applications

**Table 3:** Comparison of AML-based IoT-enabled security systems.

Ref.	Year	ML Model	Attack Setting	Attack Method	Defense Method	Pros and Cons
[18]	2020	GBM, RF,	White-box	Label modification	-	(+) Heterogeneous data sources
		NB, FDL				(−)CPU-based training
[19]	2021	DNN	Black-box	Saliency map	-	(+) Realistic threat model
				FGSM		(−) High training complexity
[20]	2023	RF, XGB,	Black-box or	A2PM	Adversarial training	(+) Regular and adversarial training
		LGBM, IFOR	gray-box			(−) Focus only on accuracy rate
[21]	2021	SVM	White-box	FGSM	NN, C4N	(+) Robust malware detection
						(−) High processing time
[22]	2021	CNN	-	GAN	A3GAN	(+) Robust malware detection
						(−) Vulnerable to inference attacks
[23]	2021	DNN	Black-box or	SGSIM, M-SGSIM	-	(+) Low time cost
			white-box			(−) Transferability of adversarial examples
[24]	2022	DNN	White-box	JSMA, FGSM,	Adversarial training	(+) An adaptive IDS and consistent adversarial training
				BIM, DF, C&W		(−) Focus only on accuracy rate
[25]	2021	SVM, ANN	White-box	FGSM	Adversarial training	(+) Use of random flip method, accuracy, and recall scores
						(−) Generation of adversarial examples in a dataset with balanced classes
[26]	2021	CNN, LSTM, GRU	White-box	FGSM	Adversarial training	(+) Adaptability to dynamic environments
						(−) Significant computational resources for training and deployment
[27]	2022	DNN	-	JSMA, FGSM	FGMD	(+) High detection accuracy and resilience to evasion attempts
						(−) Dependency on labeled data for training
[28]	2022	MLP, RF	Black-box	-	A2PM	(+) Generation of adaptive perturbation patterns
						(−) Additional complexity computational to IDS
[29]	2021	CNN	White-box	FGSM, I-FGSM, MI-FGSM	Adversarial training	(+) Enhanced model’s robustness
						(−) High computational resources for image generation and processing
[30]	2022	DL	White-box	OEA	Adversarial training,	(+) Identifying outlier behavior
					Outlier detection	(−) Creating a separate model for outlier detection
[31]	2021	DT	-	JSMA, FGSM	Adversarial training	(+) Improved model’s robustness
						(−) High computational cost
[32]	2023	RF, DT, MLP, KNN	Gray-box	JSMA, FGSM, and DF	Adversarial training	(+) Practical relevance using a realistic IoT dataset
						(−) Few scenarios in real-world IoT environments
[33]	2024	SVM, LR, MLP, RF,	Black-box	FGSM, C&W, HopSkipJump,	Feature squeezing,	(+) High accuracy rate
		and ensemble learning		and ZOO	Adversarial training	(−) High computational complexity

## Data Availability

The original contributions presented in the study are included in the article.
